# Non-classical crystallisation pathway directly observed for a pharmaceutical crystal via liquid phase electron microscopy

**DOI:** 10.1038/s41598-020-75937-2

**Published:** 2020-11-05

**Authors:** J. Cookman, V. Hamilton, S. R. Hall, U. Bangert

**Affiliations:** 1 Physics Department & Bernal Institute, University of Limerick, Castletroy, Co. Limerick Ireland; 2 School of Chemistry, University of Bristol, Cantock’s Close, Bristol, BS8 1TS UK

**Keywords:** Soft materials, Molecular self-assembly, Nanoparticles, Transmission electron microscopy, Colloids, Organic molecules in materials science

## Abstract

Non-classical crystallisation (NCC) pathways are widely accepted, however there is conflicting evidence regarding the intermediate stages of crystallisation, how they manifest and further develop into crystals. Evidence from direct observations is especially lacking for small organic molecules, as distinguishing these low-electron dense entities from their similar liquid-phase surroundings presents signal-to-noise ratio and contrast challenges. Here, Liquid Phase Electron Microscopy (LPEM) captures the intermediate pre-crystalline stages of a small organic molecule, flufenamic acid (FFA), a common pharmaceutical. High temporospatial imaging of FFA in its native environment, an organic solvent, suggests that in this system a Pre-Nucleation Cluster (PNC) pathway is followed by features exhibiting two-step nucleation. This work adds to the growing body of evidence that suggests nucleation pathways are likely an amalgamation of multiple existing non-classical theories and highlights the need for the direct evidence presented by in situ techniques such as LPEM.

## Introduction

Crystallisation is a fundamental, natural phenomenon that occurs to produce some of the most important materials in everyday life, including agrichemicals, semiconductors, geomaterials and Active Pharmaceutical Ingredients (APIs)^1–4^. The manifestation of such crystalline forms is subject to precise nucleation and growth pathways that give rise to their use in these vital applications. For example, to revolutionise the pharmaceutical industry and improve process (batch) manufacturing, continuous manufacturing has been encouraged as the state-of-art for API production due to its advantages in terms of uninterrupted production of vital drugs, minimising waste and reducing cost^5^. For API production, where the precursor can influence the end product in terms of, for example, crystal structure (polymorph selection) and thus drug efficacy, understanding the nanoscale nucleation events which determine this are paramount to adapting an API to the more streamlined approach of continuous manufacturing^6^.

One of the earliest descriptions of nucleation and growth is the Classical Nucleation Theory (CNT) and although it is somewhat oversimplified and applied exclusively to solution-based crystallisation, it remains an appropriate model for ascertaining the important aspects of nucleation e.g. free energy barriers to nucleation and material saturation requirements. Building on CNT to develop a better understanding of solution-based crystallisation, alternative theories have been developed based on interim characterisation, direct observations and ex situ experiments of a wide variety of materials. Non-classical Crystallisation (NCC) encompasses mechanisms of crystallisation that do not comply with the criteria associated with CNT, i.e. exhibiting a critical sized nucleus or supersaturated concentrations. Instead theories associated with NCC include precursor particles larger than the atomic or molecular building blocks assumed in CNT, e.g. nanoparticles, crystalline or disordered^7^. Such novel theories conforming to NCC include the Pre-Nucleation Cluster (PNC) pathway and two-step nucleation.

Currently, there is a heightened activity of academic research in the area of NCC, in particular regarding the identity and characterisation of intermediate stages of pre-crystalline processes, with a large interest in naturally occurring materials such as calcium carbonate (CaCO_3_)^8^. Much of the research on early-stage crystallisation of CaCO_3_ is applied to crystallisation pathways of other systems such as small organic molecular crystals which are currently not well understood. Vital work reported recently by Cölfen et al. has highlighted important ex situ characteristics of an intermediate stage, the Dense Liquid Phase (DLP), revealing the potential crystallisation pathway of an API, ibuprofen^9^. The DLP is an overlapping detail between PNC pathway and two-step nucleation, where D. Gebauer et al. outlined that a liquid droplet intermediate forms before solidifying into a crystalline entity^10^, and the appearance of the densified nucleus is described for protein crystallisation undergoing two-step nucleation^11^.

Full characterisation and identification of the DLP, of all materials that undergo this intermediate restructuring phase, will provide key information on what external parameters affect the materialisation of the end product. For materials that can undergo polymorphism, it is considered a general rule that prior to crystallisation of the end product, Ostwald’s rule of stages (OSR) will apply^12^. This rule details that the route taken for the formation of a final lowest energy, and therefore most stable, crystalline state will first transition through other less stable states in order of stability. However, OSR does not always pass through all stages, as has been recently rationalised for CaCO_3_ as a material susceptible to polymorphic transition^13^. Therefore, whether the intermediate state is primarily amorphous or crystalline, or composed of transient metastable polymorphs, its identification and further characterisation will give access to vital information surrounding the materials’ crystalline state prior to its final, stable crystal formation. This is especially important for APIs which are commonly polymorphic, where properties such as solubility and bioavailability are associated with the crystal structure of the active compound. If previously inaccessible polymorphs (with potentially more desirable properties) are present in the intermediate stages of crystallisation this opens avenues to harness and potentially direct their formation.

Techniques that can enable direct observation of early-stage crystallisation are needed for an in-depth understanding of API crystallisation which will extend to other small molecular crystals.

Herein, we report the nanoscale, early-stage crystallisation events of a common API, flufenamic acid (FFA), classed as a Non-Steroidal Anti-Inflammatory Drug (NSAID), targeting the enzyme responsible for inflammatory pain, i.e. cyclooxygenase (COX)^14^. FFA is freely soluble in ethanol and other low polarity alcohols, however, it is poorly soluble in water. The efficient bioavailability of an API in aqueous solutions is linked to its performance as an orally administered drug^15^. Considering this, if a drug is poorly soluble in the aqueous environment of the body, there will be low bioavailability leading to ineffective treatment of the ailment and thus requiring higher or more frequent doses, increasing the possible undesirable side effects.

For Liquid Phase Electron Microscopy (LPEM) experiments, FFA has been a facile system to induce nucleation by exploiting the radiation chemistry effects resulting from the electron beam interacting with the solvent. The authors have reported this previously by subjecting a 50 mM solution of FFA in ethanol to electron beam irradiation to produce hexagonal FFA crystals, indicative of the simulated and expected morphology of form I, used in the formulation of the commercialised NSAID^16^.

Encompassing the background of the FFA molecule and leading nucleation theories, the challenge remains to rationalise, with the aim to gain control over, the precise nucleation events that produce efficacious drugs^16^.

Towards this aim, LPEM is used to provide access to direct visualisation of the early-stage nucleation events that can provide evidence for particular nucleation and crystallisation pathways. Previous reports using LPEM have revealed new information about the growth processes of inorganic materials and biominerals leading to new theories on how these processes can be controlled or rationalised^17,18^. For instance, regarding the sintering of metal nanoparticles, in situ TEM has been used to suggest that the mechanism involved is in fact a migration and coalescence of particles followed by Ostwald Ripening (OR)^19^. More recently, Zhaoming Liu et al. revealed that the crystallisation transformation from a transient amorphous CaCO_3_ calcite occurred in the absence of morphological alterations. They concluded that the transformation process was dominated by dissolution-reprecipitation, in contrast to previous reports which lacked the in situ direct LPEM observations, which suggested direct transformation^20^.

Direct imaging of PNCs through cryo-TEM has been reported to disclose the crystallisation process of organic aromatic compounds in their near-native environment^21^. This progressive study gave insight into the isolated stages of the pre-nucleation events leading to an eventual molecular crystal form. However, due to the fragmented snap-shot evidence of the PNC stages, there is a lack of information of the intermediate steps which can provide information about the mechanism of evolution into the final crystalline stage, specifically, whether a molecular cluster evolves into a stable aggregate from an amorphous state.

Gaining access to the intermediate steps in nucleation processes is central to relating the well described NCC theories to directly observed particle dynamics and formation. The gathered knowledge will also apply to all fields which depend on understanding the nucleation of small organic crystals e.g. catalysis and agrichemicals.

Using LPEM, we probe the nanoscale events involved in the formation of crystalline entities of FFA crystals. The observed phenomena are directly linked to similar observations in other materials and the NCC theories outlined above.

Herein, we report the first direct observations of the nanoscale intermediate nucleation pathway events of a small organic molecular crystal commercialised as an NSAID, FFA, in an organic solvent.

## Results

### Radiolysis considerations

There are two general approaches towards the unavoidable beam interactions; mitigating the effects or exploiting them. High fluence has been reported to damage organic crystals in a conventional TEM setup^22^, however, the high input energy from the electron beam can also induce nucleation and enable crystal growth by radiolysis of the solvent^23^. The beam effects can be reduced by minimising the dose absorbed by the sample by using a low-dose rate and restricted exposure time; radiolysis can also be mitigated by providing a constant flow of undisturbed solvent through the liquid cell^24,25^. Herein we use what is considered to be a high dose of > 150 e^−^/Å^2^/s to ensure nucleation would occur.

In previous work by the authors, hexagonal FFA crystals were grown from a 50 mM solution of FFA in EtOH. It was hypothesised that radiolysis of the solvent by the electron beam played a vital part in allowing FFA to partake in nucleation in an undersaturated solution^16^. In the aforementioned experiments there were no pre-formed particles present to induce nucleation. It was hypothesised that the break-up of EtOH molecules due to radiolysis and the subsequent formation of reactive species altered the local chemical environment to lower the energy barrier sufficiently for the FFA molecules to nucleate.

In the LPEM experiments reported herein, it is hypothesised that the radiolysis events and resultant products could act to influence the chemical system by catalysing the early-stage nucleation events, i.e. influencing the timescale of such events to be potentially more rapid than in typical saturated solution experiments.

### Primary observations

Initially, there were no features of note in the 30 μm^2^ viewing area of the silicon nitride window at the onset of the experiment. To induce nucleation, the electron beam was condensed using the monochromator, hence increasing the electron flux in the illuminated region, but in this instance this method resulted only in scarring of the silicon nitride window. Troubleshooting methods were followed however, the lack of crystallisation events and consequent scarring of the silicon nitride windows was indicative of solution absence between the windows (see “Methods” section on Troubleshooting LPEM experiments). Subsequently, water was introduced to the syringe pumping system to primarily dislodge and carry particulates suspected to be trapped in the PEEK tubing lines of the holder, chosen because FFA is practically insoluble in water (0.008 mg/mL compared to 245 mg/mL for ascorbic acid better known as Vitamin C)^26,27^.

However, it is a possible consideration that water in these experiments could act as an antisolvent, altering the saturation conditions for the FFA molecules in EtOH. Although there was no evidence of this, unmonitored nucleation of the observed particles could have happened. From the following observations we consider that it is unlikely to have occurred, as the process would have continued in the viewing area and likely have been accelerated by the electron beam induced effects.

**Figure 1:**
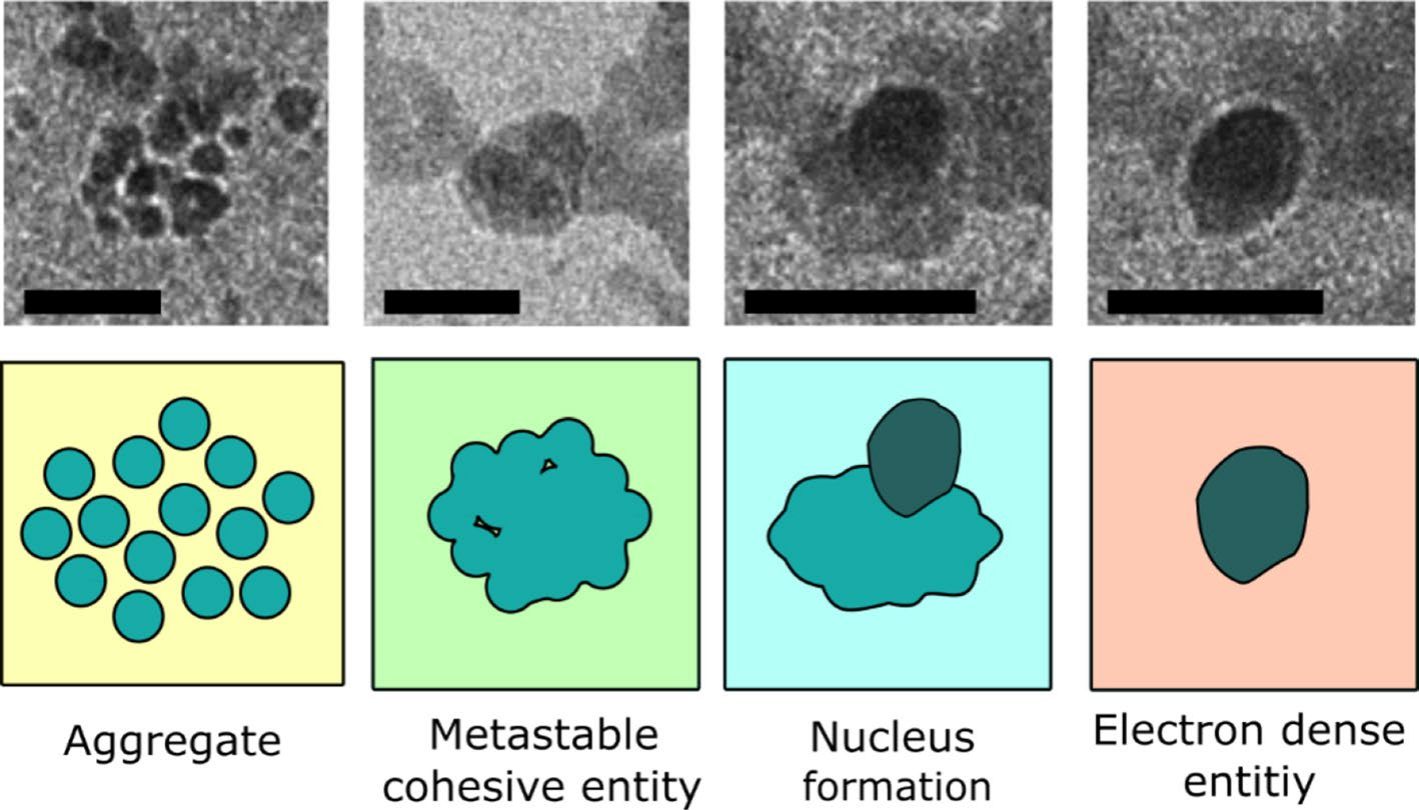
A summary of the observed processes involved in the nanoscale crystallisation of FFA; aggregation, coalescence, nucleus formation from the densified intermediate material and, finally, crystallisation. The processes are linked with NCC mechanisms such as PNC pathway and two-step nucleation theory. Scale bars: 20 nm.

The subsequent observations of the viewing area revealed a sudden influx of aggregated electron dense particles of FFA (Figure S1), which we can presume were formed and trapped in the PEEK tubing (Fig. 1).

There were two populations of FFA particles identified of 3 nm and 8 nm (Figure S1a). The 8 nm population possessed an observed pseudospherical morphology and tended to form aggregates with different numbers of particles while the 3 nm population remained dormant. We describe these small particles as Pre-Nucleation Clusters (PNCs) since they are particles that result in the formation of a metastable new phase, as described by the PNC pathway, previously reported for the crystallisation of CaCO_3_^28^.

In the area of view there were multiple dynamic events occurring, obscuring the complete process for many aggregates, however, it was possible to closely follow three notable aggregates of PNCs (Figure S2). Each PNC aggregate had varying quantities of particles upon first observation but underwent simultaneous transformational events synonymous with NCC. These transformation stages corresponded closely with PNC pathway and two-step nucleation, the precise details of which will be discussed in two sections:

1. Coalescence—detailing the events associated with the aggregation of the PNCs and the subsequent coalescence to form a metastable cohesive entity.
2. Densification towards crystallisation—describing the densification and development of a nucleus, evident by density fluctuation and formed by the successive sacrifice of surrounding material, leading to the formation of a new crystalline-like object, significantly more electron dense than its metastable predecessor.

### Coalescence

It was evident that the PNCs had aggregated prior to the initial observations. The primary transformation observed for the three selected aggregates was that of coalescence, where the individual PNCs of 8 nm merged into one uniform entity after around 3 min of continuous observation. For all three selected aggregates a similar timeline was observed (video S1) which could be indicative of the local environment catalysing the events, e.g. radiolysis breakdown of EtOH and/or water as a result of the electron beam.

**Figure 2:**
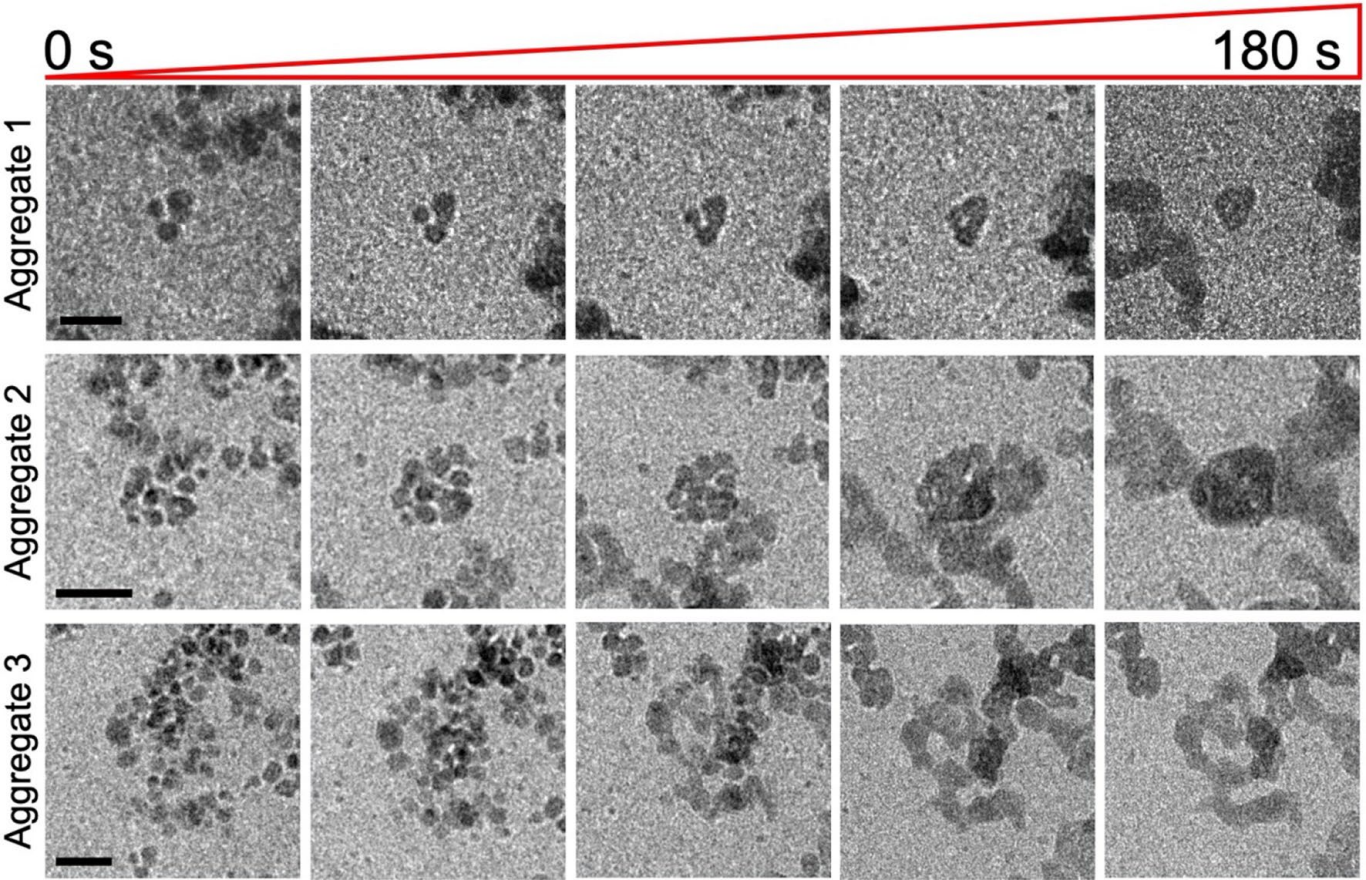
A time-lapse of each of the aggregates of PNCs undergoing coalescence. The process for the aggregated PNCs to coalesce and create a cohesive substance took 180 s, where separation between PNCs gradually reduced until there was no observable boundary between PNCs. Scale bars: 20 nm.

Each aggregate consisted of a different number of PNCs but over the period of 180 s their interfaces merged resulting in a cohesive entity notably smaller in size compared to the net size of the PNC aggregate (Fig. 2). The action of coalescence of the particle cluster has been described in a study of amorphous calcium carbonate (ACC) where Pouget et al. showed that the nucleation of nanoparticles of ACC proceeds via aggregation of PNCs in solution^28^. The formed cohesive entity can be compared to the amorphous or liquid intermediate stage according to the PNC theory, where a DLP is formed following aggregation and coalescence preceding the formation of a crystalline species^10^.

This intermediate stage has been described as a densification of the molecules in questions i.e. FFA, where the molecules are in a higher concentration compared to the mother solution. As such, in the DLP the molecules are hindered in their translational and rotational movements and kinetically stable (metastable) whilst still possessing an interface with the surrounding liquid^9^.

The individual PNCs of FFA are easily identified due to their dark appearance compared to the liquid environment, despite their small size and not possessing any metallic components. This suggests that the PNCs are crystalline (highly ordered) which may also be implied by their stability. As the PNCs coalesce into the metastable DLP, the cohesive body appears less dark compared to the individual PNCs. This radical reduction in electron density (but still easily identifiable from the surrounding liquid environment) is suggestive of a loss of structural integrity or dynamic structural change preceding the next stage of the process. It is therefore indicative that the observed intermediate phase aligns well with the explanation of PNC theory where a metastable DLP intermediate is formed prior to crystallisation.

The observed process leading to the coalescence of PNC aggregates is akin to the PNC pathway where precipitation of a new phase is evident. Although the individual PNCs created appropriate diffraction contrast in TEM conditions to suggest that they are structured or even crystalline, SAED could not be completed due to the thickness of the entire specimen arrangement, which the electron beam has to traverse in LPEM. The following transformation from the suggested DLP towards densification, however, provides important information on the formation of this API drug crystal and potential polymorph transitions.

### Densification towards crystallisation

**Figure 3:**
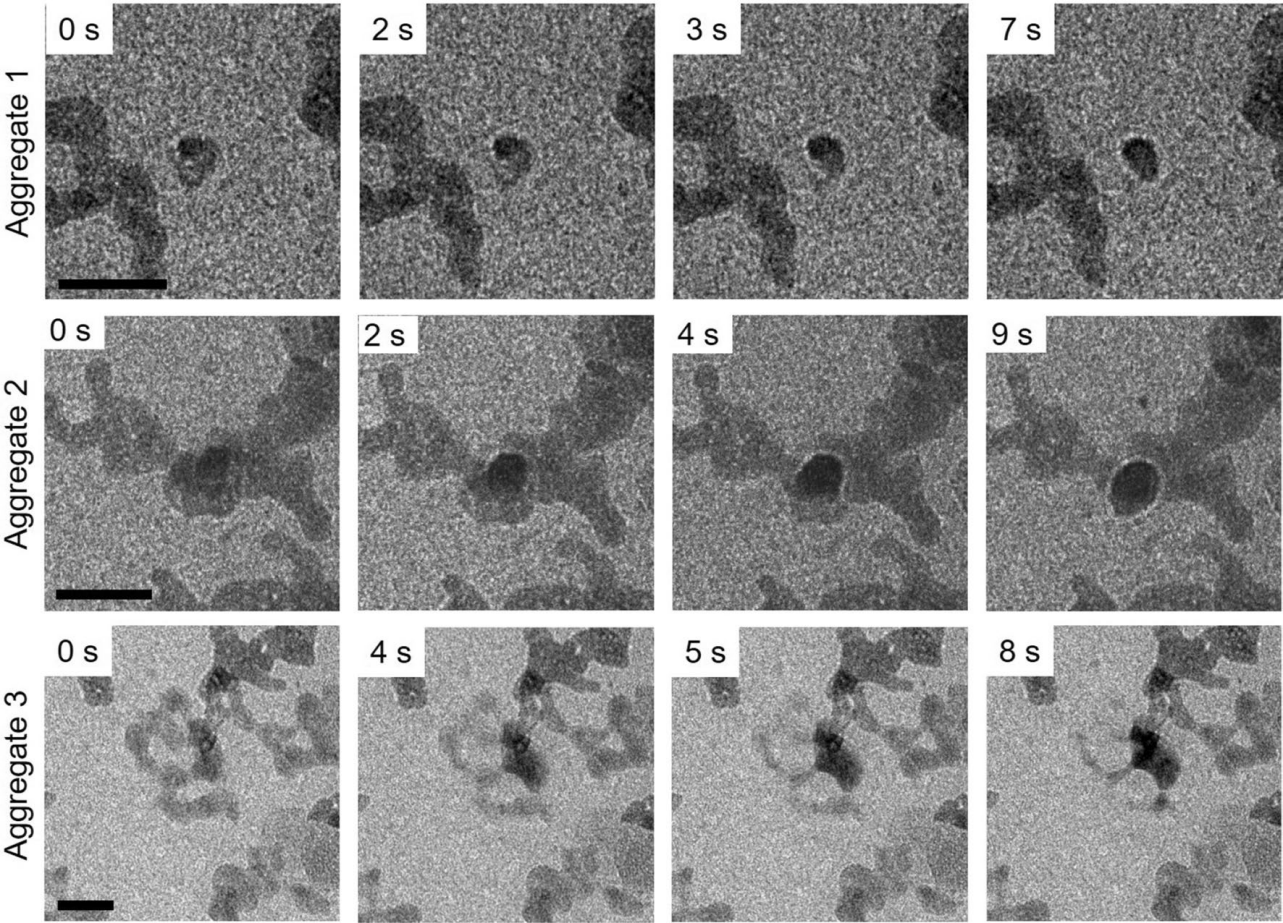
A frame-by-frame summary of the three aggregates illustrating the pre-crystallisation process of FFA: an electron dense nucleus develops and then consumes the material in the metastable cohesive entity until a final electron dense entity was formed. Scale bar: 20 nm.

In all three examples, following the cohesion of the individual PNCs they remained as a DLP until there was an apparent fluctuation, which resulted in a nucleus with a darker contrast. In TEM imaging, the darker contrast can be due to mass/thickness or diffraction contrast. Hence, the appearance of the darker nucleus, can be due to the fact that a denser and/or more crystalline phase is formed at the expense of the cohesion of the PNCs. Due to the small and rather similar size of the particles, it can, however, be assumed that they are similar in composition (the tri-fluoring functional group being the more electron dense component) and structure. Hence, the darker contrast is most likely due to diffraction (arising from an ordered phase) of particles with a certain orientation with respect to the electron beam. Therefore, it can be inferred that the darker particles (Fig. 3) are more ordered/crystalline than the surrounding and preceding phase, which is lighter in contrast, and potentially also only partially crystalline.

In a short period of time, within 10 s, densification was apparent, and the nucleus grew larger while the surrounding material in the metastable DLP appeared to be consumed to facilitate this (Fig. 3 and Videos S2, S3 and S4). The observed densification or solidification is akin to both PNC pathway and two-step nucleation. On PNC pathway D. Gebauer et al. outlined that a liquid droplet intermediate forms before solidifying into a crystalline entity^10^, and the appearance of the densified nucleus is reported for protein crystallisation undergoing two-step nucleation^11^. It is indicative therefore that the crystallisation pathway of this API (and also others) follows its own crystallisation pathway with events akin to reported non-classical crystallisation pathways.

As the nucleus grows larger the electron density increases drastically indicating that a more ordered, perhaps crystalline body had formed from the DLP intermediate. In LPEM there are challenges associated with the thickness of the liquid layer affecting image and video acquisition and resolution. Thus, acquisition of crystallinity confirmation through lattice resolution imaging or Selected Area Electron Diffraction (SAED) was not possible throughout the observed crystallisation process. The difficulties associated with resolution limitations in LPEM are discussed thoroughly by de Jonge et al. and also highlighted in a previous paper by Cookman et al. specifically when imaging FFA^16,29^.

The act of material consumption can be related to Ostwald Ripening (OR). All 3 aggregates (Fig. 3) show this clearly, where the material in the immediate vicinity of the nucleus is first consumed followed by the rest of the entity forming a new ovular particle. Interestingly, although the event shows the consumption and densification of the DLP, the other particles in the area are unaffected by this activity suggesting that they do not have the thermodynamic driving force to urge involvement with nucleus generation and further OR.

The timescales of the individual events varied notably; coalescence proceeded over up to three minutes while the transformation from DLP to formation of an electron dense entity was rapid, happening in under five seconds. As we consider the effects of radiolysis on these experiments it is possible that these timescales are influenced by the accumulative electron fluence incident on the sample. The constant electron beam penetration contributes to the production of radiolytic products such as O_2_ gas (causing oxidation), H^+^ and H^.^ (altering the pH) and other molecular products contributing to the local chemical environment of the events we report^16,30^.

**Figure 4:**
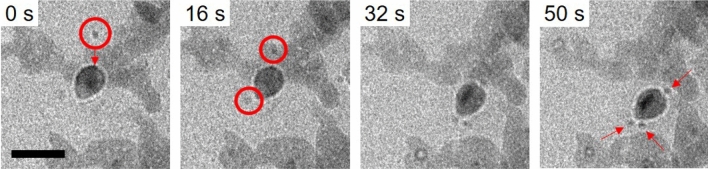
A time-lapse of the electron dense entity formed from aggregate 2 with smaller particles moving rapidly towards it (red circles). After one minute three satellite particles of ~ 3 nm were seen surrounding the new particle. Scale bar: 20 nm.

Once the electron dense entity had fully developed, aggregate 2 (Fig. 4) developed a halo-like white ring around its boundary. It is likely that during the transformation process, the particle changed z-height and the white halo is symbolic of the crystal being under-focussed compared to the DLP. Prior to the crystallisation events of aggregate 2, the aggregate appeared static and strongly interacted with the silicon nitride substrate. This strong interaction has previously been reported and therefore, the halo-like white ring is likely to be a result of defocus due to the dislodging of the particle^31^. It is also possible, that there is some interfacial interaction of the surface and the surrounding liquid, this may be the creation of a solvation layer effect or surface charging. However, reported solvation layers have been suggested to be ordered which would instead increase electron density and so the mechanism behind this requires more comprehensive investigation which is beyond the scope of this report^32^.

Immediately after the potential crystallisation event it was noted that small particles of 3 nm were rapidly attracted to the new, presumed crystalline, particle of aggregate 2 (Fig. 4 and Video S5). It was expected that the action would result in OR however for the duration of observation, the newly formed aggregate was stable, the larger particle did not appear to grow and the small particles did not behave sacraficially.

There was a density fluctuation within the crystal resulting in the formation of a dark nucleus becoming denser from 32 s onwards. This nucleus is similar to initial densification (Fig. 3) and like the initial 8 nm PNCs, the surrounding particles are stable and do not partake in OR by contributing to the growth of the crystal. It can be hypothesised that this new aggregate could undergo the same NCC process of cohesion and subsequent crystallisation. Due to the presence of the surrounding particles it is possible that some are underneath the crystal generating mass-thickness effect creating a darker feature.

## Discussion

In summary, we report that the nanoscale crystallisation of FFA occurs initially through PNC pathway evidenced by the existence of pre-formed particles of ~ 8 nm. These PNCs were a result of unmonitored nucleation that was suspected to have occurred due to the introduction of an anti-solvent, water, which subsequently carried the pre-formed particles to the area of view. The PNCs aggregated, coalesced and formed a DLP which underwent densification forming suspected crystals of FFA. In reporting these observations, a number of considerations were noted to add to the complexity of interpreting these observations. Namely, crystallinity could not be confirmed of the PNCs or the final particles through SAED. However, the contrast is most likely diffraction contrast suggesting that they are highly ordered and potentially crystalline. Due to the nature of LPEM, the monitored aggregates were in the vicinity of other particulates of FFA. Although they did not interact with the reported events, it is possible they could influence the local chemistry and therefore the interpretation of the imaging. Despite these limitations, the observations reported clearly follow NCC and it can be concluded that the observed nucleation and crystallisation pathway for this small organic molecule aligns well with both PNC pathway and two-step nucleation.

Upon coalescence of the PNCs, the intermediate species of FFA were smaller, measuring ~ 11 nm for aggregate 1 and ~ 18 nm for aggregate 2 (8 nm PNCs). However, there is no immediate comparison of the intermediate phase sizes when in the case of the reported FFA, this was directly related to the number of PNCs in each aggregate. It can be suggested that the size of the intermediate phase can be estimated by the number of PNCs on the aggregate.

The exact characterisation of the composition of the DLP remains challenging particularly in the absence of SAED, which would allow for crystalline phase identification along with that of the preceding and following phases, PNCs and post-densification, allowing for a thorough comparison to be made. Building on previous characterisations of the intermediate from PNC and two-step it is hypothesised that, in the case of FFA, the intermediate is a DLP and can be assumed to be somewhat amorphous.

This structure has been described as highly dynamic, containing ionic polymers with dynamic topology consisting of chains, branches and rings of the associated polymer. The dynamic environment reported for ACC can rapidly exchange with the ions in solution but are an independent formation and thermodynamically stable at mM concentrations.

However, due to the polymorphic characteristic of FFA it can be rationalised that Ostwald’s Rule of Stages (OSR) can occur. To develop the hypothesis, it must instead be assumed that the cohesive structure is poorly crystalline (disordered) and/or contains regions of amorphous material to account for the poor electron density compared to the PNCs (Fig. 1). OSR states that a material susceptible to polymorphism undergoes transitions through high energy polymorph states before crystallising as the low energy accessible polymorph. This can occur as a waterfall transition where the high energy state is reached and continually transforms to the next available lower state. OSR has been reported for crystallisation of CaCO_3_ where it cascades through the polymorph forms of vaterite and argonite before reaching the ground state to crystallise as calcite^13^. Considering this, it is possible that the DLP observed is the formation of a poorly crystalline high energy polymorph (for FFA there are nine reported), before crystallising as a nucleus of a more thermodynamically stable form i.e. form I or III^16,33^.

In these experiments, we consider radiolysis of the solvent to play a part in the crystallisation events where the reactive products can catalyse and accelerate the events. The resultant ionic species expected to be produced from continuous radiolysis events are cations, protons, anionic forms of FFA, along with other radical and molecular species^16,30^. An associated theory originating from reported results of ACC, describes the intermediate phase as a structural configuration called dynamically-ordered liquid-like oxyanion polymer, aka. DOLLOP^34^. The oxyanion of FFA, as a result of radiolysis events, could partake in the DOLLOP formation, perhaps due to the anionic form of the FFA molecules as they attempt to restructure. Metastable DOLLOP formation could be why we do not observe, at this stage, defined hexagonal crystals indicative of form I FFA^16^.

A similar observation akin to DOLLOP and DLP was highlighted in the recent study by Cölfen et al., where the metastable liquid phase of ibuprofen crystallisation was said to consist of a high concentration of ibuprofen molecules with hindered molecular dynamics.

The DLP intermediate has overlapping characteristics with the primitive nucleation events of protein crystals according to two-step nucleation where a metastable dense liquid develops a densified nucleus prior to crystallisation^35,36^. With regard to FFA, we identified that the intermediate phase following aggregation of PNCs, forming a DLP as described in PNC theory, is also described by the first stage of two-step nucleation theory, compounded by the formation of a dense nucleus. These findings suggest that these two NCC theories can be complementary describing different stages of the pre-crystalline process.

**Figure 5:**
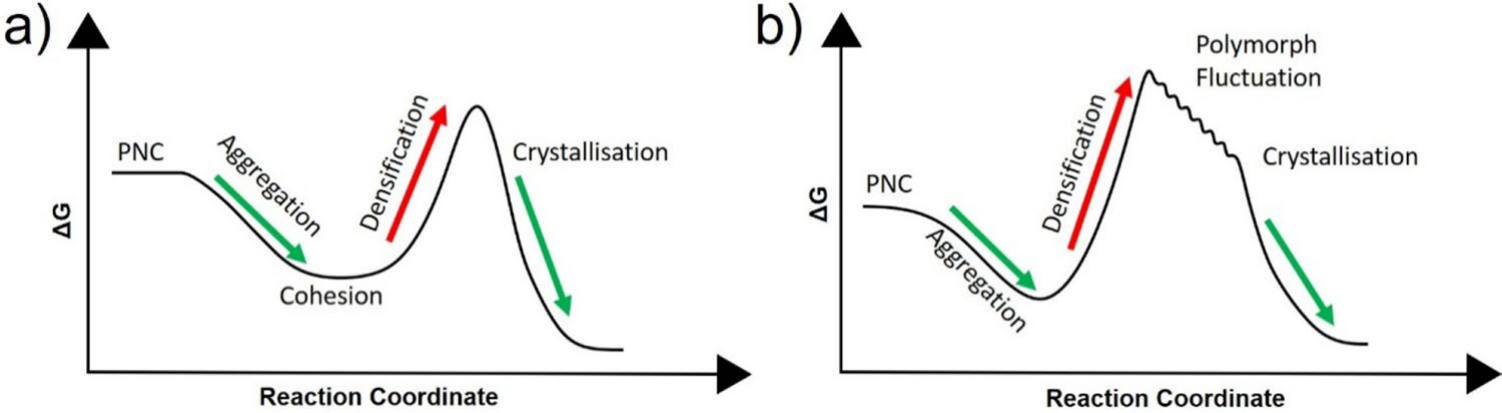
An illustration of two proposed energy diagrams of FFA based on the observations presented and knowledge of energy requirements. (**a**) An interpretation of the energy diagram following PNC pathway indicating the energy requirements for each process of PNC aggregation, cohesion, densification and crystallisation. (**b**) An illustrated energy diagram considering the hypothesis that following cohesion, the DLP consists of a fluctuation of high energy polymorphs (according to OSR) until crystallisation of a low energy, more stable polymorph of FFA. Inspiration for these energy diagrams were gathered from reports on energy requirements for NCC^37,38^.

From available literature surrounding potential energy barriers to formation, we propose two potential pathways which both possess energy requirements for each stage to progress and for the interfaces to be generated (Fig. 5). In both cases, the PNCs aggregate reducing the interfacial energy between the individual PNCs by creating a lower energy aggregate. Cohesion of the PNCs takes place forming metastable entities in both scenarios; however, the structure of this entity is proposed to be composed of two starkly different bodies in terms of energy requirements. The first proposal is that the PNCs form a disordered or DLP, behaving like an amorphous body, where relaxation of the energy occurs prior to the energy barrier of densification. The second aligns with OSR where the metastable body is composed of FFA molecules whose movements are hindered, are then able to dynamically interchange between high energy polymorphs in a stepwise fashion. The thermodynamically stable polymorph (lowest in energy) forms with a relaxation of energy but a subsequent energy penalty where crystallisation of all available material occurs.

Although many of the first pivotal reports on PNC pathway were borne from investigations on ACC and other CaCO_3_ derivatives, it can be regarded that other materials such as small organic molecules may not follow these exact pathways. Taking into account the most prominent and widely accepted crystallisation theories reported to date, it is therefore likely that FFA undergoes crystallisation via NCC displaying a close comparison to the PNC pathway followed by a likeness to two-step nucleation.

These direct observations reported for the crystallisation of FFA reveal compelling new information regarding the possible pathways of the nanoscale nucleation and intermediate events preceding the formation of the crystalline API.

From observations reported in this manuscript, it is clear that FFA undergoes a nonclassical nucleation and crystallisation pathway that predominantly follows the pre-nucleation cluster pathway due to the presence of small clusters. Upon formation of the intermediate the crystallisation pathway then diverts to two-step nucleation where densification of the intermediate resulting in nucleus formation dominates.

To conclude, it can be presumed from previous observations of the system that FFA begins as independent PNCs of approximately 8 nm in size which then aggregate. Three independent aggregates of PNCs were monitored as they separately underwent coalescence into a metastable phase described as related to the dense liquid phase (DLP) in PNC. From this point, features of two-step nucleation were identified with nucleus formation in the DLP, typically associated with protein crystallisation. Densification of the DLP forming a nucleus displayed characteristics of Ostwald Ripening (OR) where the DLP material was consumed at the expense of the new more ordered structure concluding the observed crystallisation process.

FFA is a low-electron dense organic molecule suspended in an organic solvent, the high electron densities observed for the PNCs and also the final entity infer that there is structural ordering towards crystallinity offering this advantage over the surrounding liquid. Tracking of the electron density fluctuations throughout the crystallisation process was used to identify stages in the individual pathway of FFA crystallisation from PNC aggregates. The stages proposed are PNC identification, aggregation and coalescence of PNCs into a DLP (or high energy polymorphic transition), nucleus formation through densification and finally crystallisation. However, in interpreting the data it was clear that the crystallisation process displayed did not conform completely with one theory but instead echoed certain parts of a multitude of theories and mechanisms i.e. PNC, OR, two-step nucleation, DOLLOP etc.

The work presented demonstrates that LPEM is a required and complementary tool to probe chemistry at the nanoscale, in particular the nucleation of APIs, where control at the molecular building block stage can be of direct benefit for the production of the end product. In the undersaturated conditions required for use in the liquid cell, a more controlled environment can be achieved to establish the precise stages of nucleation whilst directly observing each event in succession, as we have shown.

This developing topic regarding the direct visualisation of nucleation of pharmaceutical products will provide the necessary information to further refine industrial-scale processes by outlining the crystallisation pathways that small organic molecular crystals take to better streamline production activities and develop continuous manufacturing processes for generic drugs.

Much like the discovery of NCC had superseded CNT in practical circumstances, we find that a more complex crystallisation pathway is observed for the common API, FFA, than that described by one singular NCC theory. This indicates the complexity of controlling the nucleation and resulting crystallisation of many small organic molecules, of particular importance, resulting in APIs, highlighting the real need for continued investigation by direct means for a complete understanding of this process.

## Materials and methods

### Materials

Flufenamic acid (FFA) (Sigma Aldrich, 97%), ethanol (Fisher Scientific, HPLC grade), water (Fisher Scientific, HPLC grade).

### 5 mM flufenamic acid solution preparation

In a glass vial FFA crystals were weighed out and dissolved in the appropriate amount of ethanol to result in the preparation of a stock solution of a higher concentration of FFA e.g. 50 mM. Ethanol was filtered using a 0.2 μm PTFE filter (Fisher Scientific) fitted to a 10 mL syringe. Filtered ethanol was introduced to the FFA crystals in the glass vial and the vial was inverted several times to ensure full dissolution of the crystals. The stock solution was filtered using an 0.2 μm PTFE filter fitted to a syringe and then a 1:10 solution was prepared from this using filtered ethanol, resulting in a 5 mM concentration of FFA.

### Liquid cell TEM holder preparation

The Thermo Fisher Scientific (TFS) compatible DENSsolutions Ocean holder (H-SL-FS-005) was assembled with windowless silicon chips (DENSsolutions) to test the vacuum tightness of the O-rings. The assembled holder was placed in a vacuum pump (Pfeiffer vacuum, HiCUBE ECO), once the vacuum reduced to 9 × 10^–6^ mbar in under 10 min the O-rings used in the assembled holder were deemed suitable and capable of vacuum sealing. The holder was then plasma cleaned (Gatan Solarus) for 15 min with a combination of H_2_ and O_2_ to remove any carbon contamination that could affect the vacuum and outgassing of contaminants in the TEM column.

### Silicon chips preparation

A pair of silicon chips (Nano-Cell, Si_3_N_4_ windows 400 μm × 30 μm, no spacer, DENSsolutions) were placed in a glow discharge (easiGlow Glow Discharge Unit, 30 W, 10 mA) chamber and subjected to glow discharging (air) for 5 min. To assemble the LCEM holder, the first chip was placed in the bottom of the holder and the second chip was placed upside down (so the windows faced each other), orthogonally. The holder underwent another leak test procedure (pressure to reduce to 9 × 10^–6^ mbar in under 10 min) to ensure the windows were not damaged in the cleaning or assembly process.

### Liquid phase electron microscopy

The follow method for LPEM setup has previously been reported by the authors^16^.

Liquid Phase Electron Microscopy was conducted using a TFS (formerly FEI) Titan Themis^3^ operating at an acceleration voltage of 300 kV, fitted with a monochromator. The TEM was operating in conventional parallel beam TEM mode using an objective aperture for image and video acquisition. Data was acquired using a Gatan OneView (4 k x 4 k).

Alignments for the TEM were completed with a gold standard sample on a conventional single tilt holder to achieve optimal resolution, these include refining the image corrector and setting the monochromator to achieve controllable low dose conditions. Once the alignments were complete, the Ocean holder was inserted into the TEM undergoing a 10 min pre-pump before fully inserting the holder into the column. The PEEK tubing was connected to a solution of filtered ethanol in a 3 mL syringe fitted to a syringe pump. EtOH was flowed through the holder at a rate of 5 μL/min for 1 h to ensure total immersion of the solution between the Si_3_N_4_ windows. The windows were viewed in ethanol only conditions to map any defects and ensure the cleaning procedure was effective^16^. Finally, a 5 mM solution of FFA was placed in a 3 mL Luer lock syringe and connected to the PEEK tubing of the Ocean holder. A syringe pump was used to flow at a rate of 5 μL/min for 1 h prior to experimental imaging. To ensure nucleation events would be initiated an electron dose of > 150 e^−^/Å^2^/s was used.

### Troubleshooting the LPEM experiment—liquid diffusion

In absence of notable observations where they are expected e.g. nucleation, it was supposed that the lack of solution diffusion was the cause. To facilitate solution diffusion through the silicon nitride windows a series of actions can be taken to rectify this. Briefly, the column valves were closed on the TEM, objective aperture was removed (using an objective aperture with non-TFS holders is to be used with caution as the height of the Ocean holder is different that single & double tilt holders provided by TFS) and the stage tilted e.g. + 20° while increasing the liquid flow from the peristatic pump to 20 μL/min. The holder and TEM were left in this condition for approximately 30 min and on return the actions were completed in reverse. Oftentimes this troubleshooting procedure allowed liquid to diffuse between the windows unless another issue prevented liquid diffusion i.e. trapped particles from uncontrolled nucleation. These trapped particles can be dislodged by introducing an alternative solvent that will facilitate carrying (rather than dissolution) of the particles, in this instance water was used.

### Troubleshooting the LPEM experiment—inducing nucleation

During the concerned experiments, nucleation was expected when the electron beam was incident on the sample for at least 15 s at a dose higher than 200 e^−^/Å^2^/s according to previous work carried out by the authors^16^. To induce nucleation where it has not occurred, the monochromator was condensed on an area for 10 s and then expanded to normal setup conditions. This will enable nucleation of the aforementioned 5 mM FFA in EtOH, however in absence of liquid this will ‘scar’ the silicon nitride window leading to the conclusion that there is no liquid between the windows. As per the initial steps of LPEM setup, EtOH (alone) is introduced prior to the FFA in EtOH solution. If the FFA in EtOH solution has not diffused through the windows yet and this induction procedure is followed a growing electron dense bubble will result but with no crystal nucleation. Therefore, the solution must be left to flow longer to ensure the FFA solution has diffused through the windows.

### Image and video processing

All micrographs and videos were acquired and analysed using Gatan’s GMS3 using the in-situ player accompanying the OneView Detector. Analysis of the micrographs and videos encompassed, binning, viewing, exporting into common readable files, and measurements of particle sizes and electron density.

## Supplementary information

Supplementary Video 1.

Supplementary Video 2.

Supplementary Video 3.

Supplementary Video 4.

Supplementary Video 5.

Supplementary Information.
