# Supplementary material for: Non-classical crystallisation pathway directly observed for a pharmaceutical crystal via liquid phase electron microscopy
Source: Sci Rep. 2020 Nov 5;10:19156. doi: 10.1038/s41598-020-75937-2 (PMC7644682; doi:10.1038/s41598-020-75937-2)
Supplement: Supplementary file 6 — Supplementary Information. [file 41598_2020_75937_MOESM6_ESM.docx]

**Non-Classical Crystallisation Pathway Directly Observed for a Pharmaceutical Crystal via Liquid Phase Electron Microscopy**

Authors

J. Cookman^a^, V. Hamilton^b^, S. R. Hall^b^*, U. Bangert^a^*

^a^ Bernal Institute, University of Limerick, Castletroy, Co. Limerick, Ireland.

^b^ School of Chemistry, University of Bristol, Cantock’s Close, Bristol BS8 1TS, UK.

Corresponding author contact details:

[simon.hall@bristol.ac.uk](mailto:simon.hall@bristol.ac.uk)

[Ursel.bangert@ul.ie](mailto:Ursel.bangert@ul.ie)

**
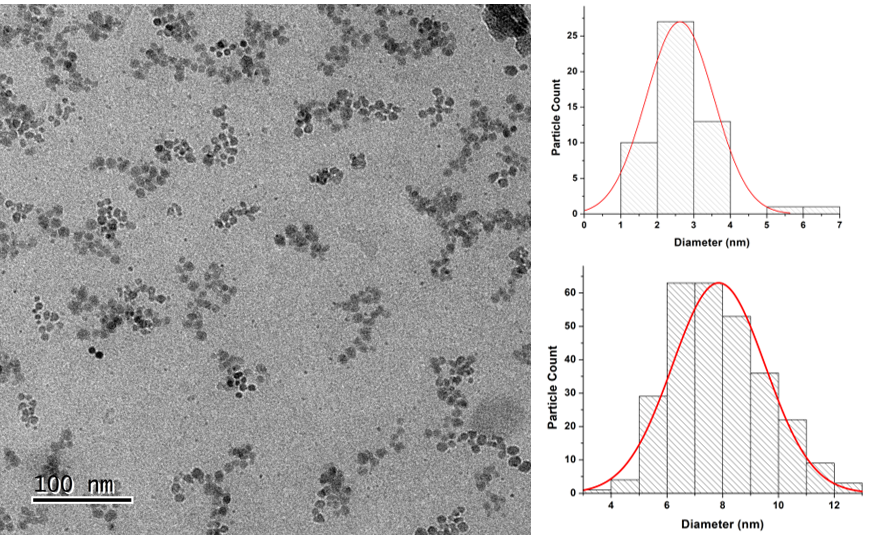
**

**Figure S1:** A micrograph of PNCs of FFA with the corresponding particle size distributions detailing that the average size of the two discrete populations of particles present of 3 nm and 8 nm.


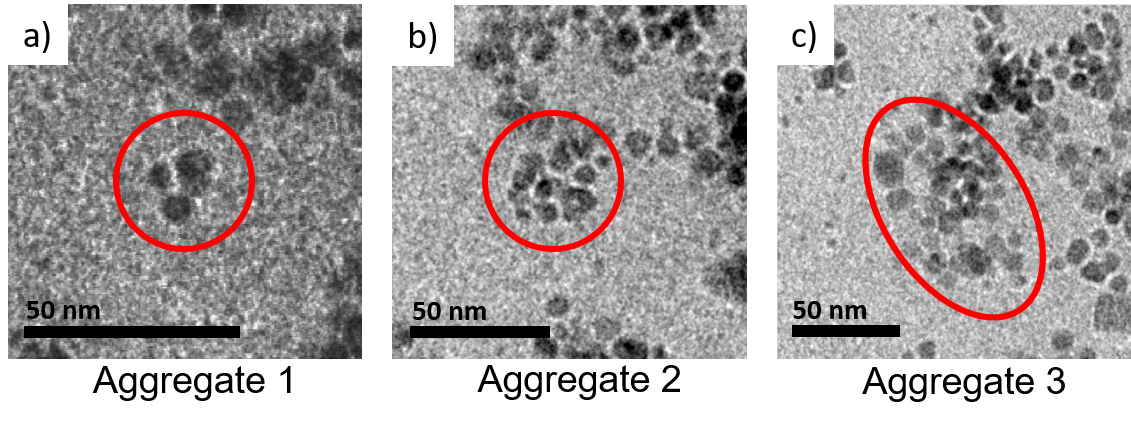


**Figure S2:** Three notable collection of particles (denoted aggregates) are monitored throughout the pre-crystallisation process, these have been named **a)** aggregate 1 consisting of four particles **b)** aggregate 2 containing ten NPs and **c)** aggregate 3 consisting of approximately 23 NP.

**Supplementary Video Files:**

**SI Video 1 Summary**

An unedited video showing the entire field of view where the 3 aggregates are identified and shown to undergo non-classical crystallisation. Indiciations in the video highlight the transformations detailed in manuscript in succession. (12 fps)

**SI Video 2.**

Aggregate 1 undergoing densification towards crystallisation (5 fps)

**SI Video 3.**

Aggregate 2 undergoing densification towards crystallisation (5 fps)

**SI Video 4.**

Aggregate 3 undergoing densification towards crystallisation (5 fps)

**SI Video 5.**

Aggregate 3 showing smaller particles attracted to new crystal and interacting 12 fps.
